# Antecedents of positive and negative intergroup contact: Evidence from a diary study

**DOI:** 10.1002/ijop.12841

**Published:** 2022-03-08

**Authors:** Francesca Prati, Sarina J. Schaefer, Miles Hewstone, Oliver Christ

**Affiliations:** ^1^ Department of Psychology, Bologna University Bologna Italy; ^2^ Faculty of Psychology, FernUniversität in Hagen Hagen Germany; ^3^ Department of Experimental Psychology, Oxford University Oxford UK

**Keywords:** Intergroup contact, Social dominance orientation, Right‐wing authoritarianism, Ingroup norms, Neighbourhood diversity

## Abstract

In our current globalised, multicultural world, understanding antecedents of reciprocal interactions between native people and people of immigrant background is a major issue, because intergroup contact plays a crucial role in building inclusive societies. In this vein, using daily diary data, we examined the relation between the number of daily positive and negative interactions of White British majority (*N* = 744) and Asian British minority people (*N* = 582) with members of the respective outgroup, with RWA, SDO, perceived ingroup norms, neighbourhood diversity and contextual deprivation. Results showed that for the majority group, ingroup norms in favour of intergroup contact were positively associated with positive intergroup encounters, whereas Right Wing Authoritarianism (RWA) was positively associated with negative intergroup contact. Neighbourhood diversity was positively associated with positive and negative intergroup encounters. Moreover, RWA moderated the relationship between neighbourhood diversity and both positive and negative contact of White British people. For the minority group, ingroup norms were positively associated with positive intergroup contact, and the relationship between ingroup norms and negative contact was moderated by SDO. Overall, different factors affect positive and negative intergroup contact of majority and minority groups. We discuss the implications of the findings for future research and interventions.

## INTRODUCTION

More than 60 years of research since Allport' (1954) original statement of the ‘contact hypothesis’ indicates that positive contact with outgroup members is associated—cross‐sectionally, longitudinally and experimentally—with less prejudice toward this group (for a meta‐analysis, see Pettigrew & Tropp, 2006). This is especially true when contact occurs under optimal conditions: equal status, cooperation, promotion of close relationships between members of the two groups and institutional support. However, intergroup contact is a more complex phenomenon than early research envisaged: it can be perceived as a positive, but also a negative experience with potentially harmful and not only beneficial consequences for intergroup relations. Whether individuals experience intergroup encounters positively or negatively is dependent on various factors. A number of antecedents of positive contact with outgroup members have been examined, mainly focusing on single variables at a time (for an overview see Kauff et al., 2021). Very few studies have examined antecedents of negative contact with outgroup members (Kros & Hewstone, 2020). Moreover, the majority of these studies focused exclusively on the perspective of the majority group. In sum, little is known about the different antecedents of negative and positive contact and the interplay between them, especially from the perspective of minority group members. In the present study, we contribute to capturing the real‐world complexity of intergroup experiences by considering simultaneously the influences of institutional, social and ideological factors and their interplay, on both positive and negative contact from the perspectives of majority and minority groups.

### Positive and negative intergroup contact

The majority of studies on intergroup contact have focused mainly on positive *or* negative experiences with outgroup members. Only recently have scholars underlined the need to examine both types of contact, as in daily settings people frequently have to deal simultaneously with positive and negative intergroup encounters (Graf et al., 2014). Negative contact might be of special importance, as negative intergroup experiences may be stronger predictors of intergroup attitudes than positive experiences (Barlow et al., 2012).

To understand the antecedents of differently valenced contact (e.g., whether the same factor that facilitates positive intergroup contact also increases or perhaps inhibits negative experiences with outgroup members) new research is needed. The antecedents tested in our research are located at the macrolevel (e.g., institutional characteristics), the mesolevel (e.g., perceived social norms) and the microlevel (e.g., ideological orientation).

### Inhibitors and facilitators of contact at the macrolevel

The role of social diversity in predicting intergroup relations and cohesion is a contentious topic. Numerous studies have shown a positive relationship between intergroup exposure and outgroup attitudes (Pettigrew & Tropp, 2006). However, among recent contributions, Laurence et al. (2018) showed that experiencing diversity positively predicted both positive and negative intergroup contact in neighbourhoods and work‐places, with the former improving and the latter harming intergroup relations. van Assche et al. (2016), using a nationally stratified Dutch sample, demonstrated that objective diversity was associated with more negative attitudes and greater mistrust toward ethnic outgroups, yet only among high authoritarians. In a recent study in the United Kingdom, including the percentage of ethnic minority group members in a neighbourhood and a composite measure of spatial segregation, Kros and Hewstone (2020) did not find these measures to be associated with negative interethnic contact. Given this complex set of findings, research is still needed to further disentangle the relationship between contextual outgroup exposure and differently valenced contact.

Another factor that could influence the amount of negative interethnic contact is the level of social and economic deprivation in neighbourhoods, municipalities, and cities (e.g., Sampson et al., 1997). Kros and Hewstone (2020) found that a composite measure of several indicators of social and economic deprivation was not associated with more negative interethnic contact for either White British or Asian British people. Nevertheless, living among increasing proportions of outgroup members is associated with more negative intergroup attitudes in communities with higher levels of socio‐economic disadvantage because of the increased threat to one's position (Laurence, 2014). This effect holds only for individuals without interethnic ties, however, suggesting a complex interlinking of the effects of diversity, disadvantage and contact. Thus, although recent studies failed to find a direct link between deprivation and negative contact, we suggest that it is worth investigating this relationship further.

### Inhibitors and facilitators of contact at the mesolevel

Ingroup and outgroup norms—shared beliefs about appropriate conduct for group members (Jetten et al., 1996)—have been shown to be powerful predictors of behaviour.

Considering both majority and minority groups, and using data from several different countries, Christ et al. (2014) reported consistent evidence that, even when controlling for individuals' own experience of direct intergroup contact, people can benefit from living in mixed settings where fellow ingroup members do engage in such contact. Similarly, in a longitudinal study with White and Asian British high‐school students, Al Ramiah et al. (2015) showed that having outgroup friends and perceiving ingroup norms in favour of intergroup contact increased the likelihood that students would indicate their willingness to sit with outgroup members in the school cafeteria. However, while pro‐contact ingroup norms facilitate positive intergroup contact, we still do not know their impact on negative contact.

### Inhibitors and facilitators of contact at the microlevel

At the microlevel, research has shown that individuals higher in prejudice report less positive contact and more negative contact relative to their more egalitarian counterparts (Dhont & Van Hiel, 2009). Three longitudinal studies consistently showed that highly prejudiced people in different countries were more likely to perceive interactions with immigrants negatively than were less‐prejudiced people (Kotzur et al., 2018). Similarly, research reliably showed that a preference for hierarchical social structures and inequality (Social Dominance Orientation, SDO; Sidanius & Pratto, 1999) as well as preference for traditional social norms, stability and order (Right‐Wing Authoritarianism, RWA; Altemeyer, 1991) independently predicted negative attitudes toward immigrants in many countries (e.g., Cohrs & Stelzl, 2010). Nevertheless, Brune et al. (2016) found that when living in areas with high proportions of immigrants, even majority group members who scored higher on RWA spent more time with immigrant friends relative to authoritarians in less ethnically diverse areas.

## OVERVIEW OF THE RESEARCH

The present research used a diary approach to investigate the joint roles of different factors in predicting daily intergroup contact, not just positive but also negative contact, for members of majority and understudied minority groups. We examined, first, the strength of the main antecedents of intergroup contact, and then their interactions in producing or inhibiting intergroup contact experiences. To realise these aims, we tracked social interactions of White British people (ethnic majority) and Asians British people (ethnic minority) living in the United Kingdom with respective ethnic outgroup members across 13 days. British Asians (largest sub‐groups: Asian British Indian 33%, Pakistani 27% and Bangladeshi 10%) account for 7% of the UK population, constitute the largest ethnic minority group in Britain (Office for National Statistics, 2013) and face discrimination across a wide range of measures (e.g., Social Mobility Commission, 2016).

We sought to extend recent research showing the role of neighbourhood diversity in predicting intergroup contact from the perspective of the majority group (Laurence et al., 2018). We hypothesized that the percentage of outgroup members in UK neighbourhoods, as an index of increased contextual opportunities for intergroup contact (Laurence et al., 2018), would predict higher frequency of outgroup contact, both positive and negative, of not only White British but also Asian British people (Hypothesis 1). Given evidence that disadvantage can be associated with negative intergroup attitudes (Laurence, 2014), we hypothesized that for both majority and minority groups multiple deprivation (based on different contextual indices of economic difficulties) would predict higher frequency of negative contact, and lower frequency of positive contact, with respective outgroup members (Hypothesis 2). We also hypothesized that ingroup norms in favour of intergroup contact, such as having family and friends who support intergroup relationships, working as a tool to promote intergroup relationships (Christ et al., 2014), would predict higher positive, but also lower negative, contact with respective outgroup members for both groups (Hypothesis 3). Extending previous research (Cohrs & Stelzl, 2010), we hypothesized that White and Asian British people higher in ideologies that are linked to ingroup threat and ethnic prejudice, such as SDO or RWA, would report more frequent negative and less frequent positive contact with the respective outgroup than those low in SDO or RWA (Hypothesis 4).

Furthermore, we explored the joint effects (i.e., interaction) of individual ideologies (i.e., RWA, SDO), perceived social norms and contextual characteristics (neighbourhood diversity, IMD) on the frequency of both types of intergroup contact. Previous research showed the role of neighbourhood diversity in increasing positive intergroup contact, especially for majority group members high in RWA (Brune et al., 2016. We extended this research by testing the moderating role of two different ideologies on the relationship between neighbourhood diversity and negative and positive intergroup contact of both majority and minority groups. We expected that among White British people high in RWA or SDO, those living in neighbourhoods with a higher percentage of Asians would experience more negative and positive intergroup encounters than those living in less diverse neighbourhoods, because it would be easier to avoid outgroup members in less diverse areas. We expected that for White British respondents low in RWA or SDO, differences between those living in more diverse than less diverse areas would be weaker (Hypothesis 5). We also expected that White British people living in a context burdened with higher social and economic deprivation would experience more negative contact with the minority group than those who live in a context with lower deprivation, especially then they were high in RWA and SDO (Hypothesis 6). Given the effective role of ingroup norms in promoting intergroup relationships (Christ et al., 2014), we tested the moderating role of ideologies in the relationship between ingroup norms and intergroup contact. We hypothesized that perceiving high ingroup norms in favour of contact would favour positive and negative intergroup encounters of White British people high in SDO and RWA (Hypothesis 7). We then explored whether these hypotheses hold for the minority sample.

## METHOD

### Participants

Respondents were recruited by Ipsos MORI survey company. Data were collected in England from March to April 2017 using smartphones, tablets, and laptops. A total of 744 majority (White British; 59.7% women, 40.1% men, 0.3% other; age: *M* = 47.32, *SD* = 15.22) and 582 ethnic minority people (Asian British; 57.9% women, 41.9% men, 0.2% other; age: *M* = 41.76, *SD* = 13.64) were sampled from mixed neighbourhoods taken from the company's online panel. The neighbourhoods were selected based on a stratified random probability sample, with the strata being defined by low, medium and high levels of economic deprivation.

All procedures performed in studies involving human participants were in accordance with the ethical standards of the University of Oxford (UK) and with the 1964 Helsinki Declaration and its later amendments or comparable ethical standards.

### Procedures and measures

Respondents were required to complete a pre‐diary survey. Thereafter, for a period of 13 days participants were asked to fill in a short diary concerning their daily intergroup encounters. We used an interval‐contingent approach (with responses completed each evening, via an app delivered to respondents' smartphone devices). We restricted the length of the questionnaire to 5 minutes/day, and paid respondents a small incentive (up to 160 points that can be traded in for online vouchers) to encourage participation.

Starting the evening of the pre‐diary survey, for 13 days participants were asked to complete a 5‐minute ‘diary‐like’ survey each evening where they reported how many contacts they had with outgroup members (i.e., Asian people for White respondents; White people for Asian respondents) during that day. Participants received links to these diaries in the evening and were asked to complete the diaries by no later than the next morning. Specifically, they were asked how many contacts they had with outgroup members (0 = none, 1 = 1, 2 = 2, 3 = 3, etc., …, 20 = more than 20). If they reported one or more contact, they were asked to indicate how many positive/good contacts and how many negative/bad contacts they had had. In this way, we assessed positive and negative daily intergroup contact.

### Pre‐diary measures were

#### Percentage of Asian people in the neighbourhood

Lower Layer Super Output Areas (LSOAs) are used by UK Office for National Statistics, designed to cover a minimum 1000 people or 400 households and a maximum 3000 people or 1000 households, to represent neighbourhoods. The percentage of Asians measure indicates the proportion of the Asian population that would have to move between LSOAs to create a completely even distribution of majority and minority groups across the larger geographical area or Middle Layer Super Output Area (MSOA) that included LSOAs. This index can be interpreted as White British people's exposure to Asians.

#### Percentage of White people in the neighbourhood

This value indicates the proportion of the White population that would have to move between LSOAs to create a completely even distribution of majority and minority groups across the larger geographical area (MSOA) that includes the LSOA. This refers to Asian people's exposure to Whites.

#### Social and economic deprivation

To assess the socio‐economic profile of the selected neighbourhoods, we used the Index of Multiple Deprivation (IMD), a UK government derived statistic that provides information on relative levels of social and economic deprivation, based on a variety of indicators (e.g., income, employment, health deprivation and disability, education skills and training, barriers to housing and services, crime, and living environment).

#### Social dominance orientation

The 4‐item Short‐SDO scale (Pratto et al., 2013) was used to assess SDO (e.g., ‘We should not push for group equality’, ‘Superior groups should dominate inferior groups’). The response scale ranged from 1 (*disagree strongly*) to 5 (*agree strongly*). After reversing two items, a composite SDO index (White *ω* = .72, Asian *ω* = .75, see Footnote 1 for *αs*)^1^ was created, whereby high scores indicate high SDO.

#### Right wing authoritarianism

We used the Authoritarianism Ultra‐Short (A‐US) scale from Heller et al. (2020), a three‐item version of the Short Scale for Authoritarianism (Kurzzskala Autoritarismus; KSA‐3; Beierlein et al., 2014) involving one item for each of the three dimensions of RWA (i.e., aggression, submission, and conventionalism). The items were: ‘People should leave important decisions in society to their leaders’, ‘Traditional behaviour should not be questioned’, ‘Troublemakers should be made to feel that they are not welcome in society’. Anchors were 1 (*disagree strongly*) to 5 (*agree strongly*). An RWA index was created; although reliability scores were less than the conventional cut‐off (White *ω* = .59, Asian *ω* = .60), they could not be improved by dropping an item.

#### Perceived ingroup norms

Three items adapted from Gómez et al. (2011) were used to assess pro‐intergroup contact ingroup norms (i.e., ‘My [ingroup] friends would consider it a positive thing to have [outgroup] friends’, ‘My family would consider it a positive thing to have [outgroup] friends’, ‘[ingroup] People in my neighbourhood would consider it a positive thing to have [outgroup] friends’). Anchors were 1 (*disagree strongly*) to 5 (*agree strongly*). Indices of perceived ingroup norms were reliable (White *ω* = .90; Asian *ω* = .91).

Other measures were included in the pre‐diary survey that we did not employ in the present study (see Appendix).

## RESULTS

### Preliminary analysis

Analyses on the frequencies of positive and negative daily contact of both White British and Asian British respondents showed that after Day 5 respondents who reported having no positive contact with outgroup members dropped out. Therefore, we did not consider the data after Day 5, and created separate indices of daily positive and negative contact by summing the number of same‐valence contacts from Day 1 to Day 5 only.

We next tested for systematic attrition between respondents who completed both positive and negative daily contact measures across the first 5 days (*N* = 499 White British; *N* = 199 Asian British) and those who did not complete even the first 5 days of the diary study, and thus were excluded from the analysis (*N* = 245 White British; *N* = 383 Asian British). Analyses revealed that White respondents who completed the first 5 days of the diary study were significantly older, were less well educated, and had a lower percentage of Asians living in their neighbourhood, than those who did not. For Asian British respondents, those who completed the first 5 days of the diary study were significantly older, were less well educated, had a higher percentage of Whites living in their neighbourhood, and scored lower on RWA. These findings show some evidence of systematic attrition, but mainly on socio‐demographic variables that were controlled for in the following analyses (see Appendix). The descriptive statistics for the pre‐diary and diary measures and zero‐order correlations between them are presented in Table 1 for both groups. A preponderance of positive over negative contact was found for both White, *t*(316) = 7.35, *p* < .001, *d* = 0.57, and Asian, *t*(50) = 7.11, *p* < .001, *d* = 1.38, samples.

**TABLE 1:** Means, standard deviations and correlations of pre‐diary and diary survey measures

	White British	Asian British							
	M(SD)	M(SD)	1.	2.	3.	4.	5.	6.	7.
1. Percentage of outgroup members	9.84 (16.29)	83.11 (21.19)	–	.32**	−.08	−.07	.13**	.35*	.22*
2. IMD	20.31 (14.71)	22.27 (15.77)	−.29**	–	−.03	−.05	−.02	−.01	.02
3. SDO	2.05 (0.71)	2.03 (0.72)	−.04	.09	–	.40**	−.35**	−.10*	.05
4. RWA	3.05 (0.80)	3.01 (0.76)	−.17*	.21**	.51**	‐	−.15**	−.06	.10*
5. Perceived ingroup norms	3.61 (0.78)	3.72 (0.79)	−.20**	.02	−.31**	−13	–	.21**	.04
6. Positive contact	8.06 (14.85)	9.87 (16.78)	−.23**	.04	−.06	.01	.31**	–	.32**
7. Negative contact	0.55 (1.83)	1.09 (6.18)	.01	.04	−.00	−.02	.15*	.42**	–

*Note*: Correlations between variables for the White British sample are displayed above the diagonal, and those for the Asian British sample are displayed below the diagonal.

IMD = index of multiple deprivation; RWA = right‐wing‐authoritarianism; SDO = social domination orientation.

* *p* < .05.

** *p* < .01.

#### Regression analyses

Although respondents were nested in different neighbourhoods and we also considered social context factors located at the neighbourhood level, we were not able to use multilevel modelling to analyse the association between the different predictors and intergroup contact. For many neighbourhoods, only one respondent was available; thus, it was not possible to decompose the variance in the variables into within and between person variance. We therefore used multiple regression models to test several antecedents of positive and negative intergroup contact and the interactions between these antecedents. We tested separately the regression models for positive and negative contact of, first, White British and, then, Asian British respondents with the respective outgroup. At the first step of all regression analyses, we included percentage of outgroup members, multiple deprivation, SDO, RWA, perceived ingroup norms and two demographic variables, age and education. We report standardised estimates of all following results.

#### White British majority sample

From the White majority group (see Tables 2 and 3), the percentage of Asians in the neighbourhood (hereafter, percentage Asians) and perceived ingroup norms were associated with a higher frequency of positive intergroup contact of White with Asian respondents (Hypotheses 1 and 3), whereas multiple deprivation was negatively associated with it (Hypothesis 2). SDO and RWA were not associated with positive contact of White respondents. Percentage of Asians and RWA were positively associated with frequency of negative contact of White respondents (Hypotheses 1 and 4). Among demographic variables, for White respondents age was negatively related to both positive and negative intergroup contact.

**TABLE 2:** Regression analyses: Summary of factors predicting positive contact of White people with Asian people

	Step 1	Step 2a (RWA)	Step 2b (SDO)
Variable	β	β	β
Age	−0.12*	−0.12*	−0.12*
Education	0.05	0.06	0.06
Percentage of Asians	0.31**	0.32**	0.32**
IMD	−0.12*	−0.10*	−0.12*
SDO	−0.01	−0.02	−0.02
RWA	0.02	0.01	0.02
Perceived ingroup norms	0.17**	0.16**	0.17**
Percentage of Whites × ideology		0.10*	0.05
IMD × ideology		0.04	−0.01
Ingroup norms × ideology		0.04	−0.06
*R* ^2^	.18		
Adjusted *R* ^2^	.17		
*F* for change in *R* ^2^	13.55**	10.28**	9.70**

*Note*: Step 1 includes all main variables; Step 2a includes RWA as ideology in the interaction terms; and Step 2b includes SDO as ideology in the interaction terms.

IMD = index of multiple deprivation; RWA = right‐wing‐authoritarianism; SDO = social domination orientation.

* *p* < .05.

** *p* < .001.

**TABLE 3:** Regression analyses: Summary of factors predicting negative contact of White people with Asian people

	Step 1	Step 2a (RWA)	Step 2b (SDO)
Variable	β	β	β
Age	−0.09	−0.09	−0.09
Education	0.08	0.09	0.08
Percentage of Asians	0.19**	0.19**	0.19**
IMD	−0.05	−0.04	−0.05
SDO	0.02	0.01	0.01
RWA	0.13*	0.12*	0.13*
Perceived ingroup norms	0.01	0.00	0.02
Percentage of Whites × ideology		0.18**	0.07
IMD × ideology		−0.05	−0.10*
Ingroup norms × ideology		0.07	−0.07
*R* ^2^	.08		
Adjusted *R* ^2^	.06		
*F* for change in *R* ^2^	4.99**	6.34**	4.39**

*Note*: Step 1 includes all main variables; Step 2a includes RWA as ideology in the interaction terms; and Step 2b includes SDO as ideology in the interaction terms.

IMD = index of multiple deprivation; RWA = right‐wing‐authoritarianism; SDO = social domination orientation.

* *p* < .05.

** *p* < .001.

In the second steps of our regression analyses, we tested separately the interaction between RWA (Step 2a) and SDO (see Step 2b) with percentage of outgroup members, IMD, and perceived ingroup norms, because of the strong correlation between RWA and SDO (see Table 1). Likewise, the variables were centred before building the interaction terms to avoid problems of multicollinearity.

**Figure 1:**
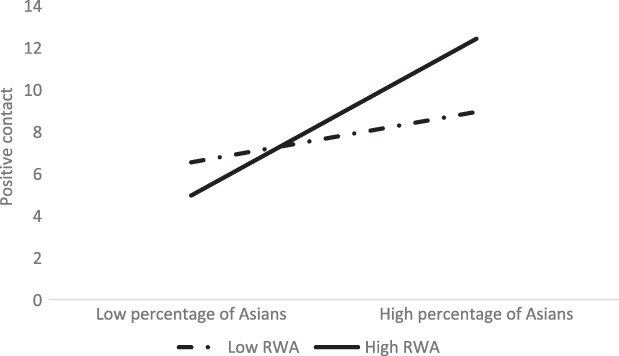
Interaction between percentage of Asians and RWA on positive contact of White people.

The interaction between percentage of Asians in the neighbourhood and RWA was positively associated with both positive and negative contact (Hypothesis 5; see Table 2). Consistent with previous research (Brune et al., 2016), and our hypothesis, the percentage of Asians in the neighbourhood was positively associated with positive intergroup contact only for White people high in RWA, *β* = 0.42, 95% CI [0.24, 0.49], but not for those low in RWA, *β* = 0.12, 95% CI [−0.02, 0.26] (Figure 1).

**Figure 2:**
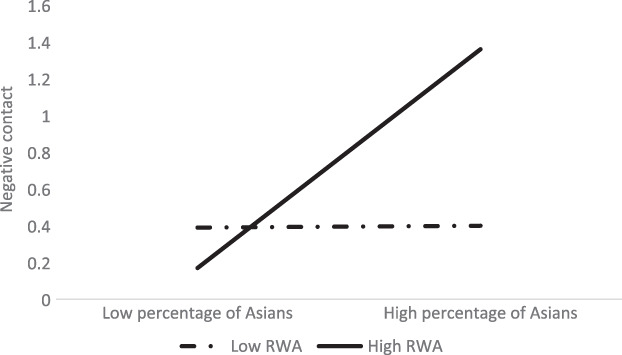
Interaction between percentage of Asians and RWA on negative contact of White people.

Similarly, the percentage of Asians was positively associated with negative intergroup contact for White people high in RWA, *β* = 0.38, 95% CI [0.03, 0.06], but not for those low in RWA, *β* = −0.01, 95% CI [−0.02, 0.01], indicating that White people high in RWA reported not just more positive but also more negative contact when they lived in neighbourhoods with a high percentage of Asian people (see Figure 2).

**Figure 3:**
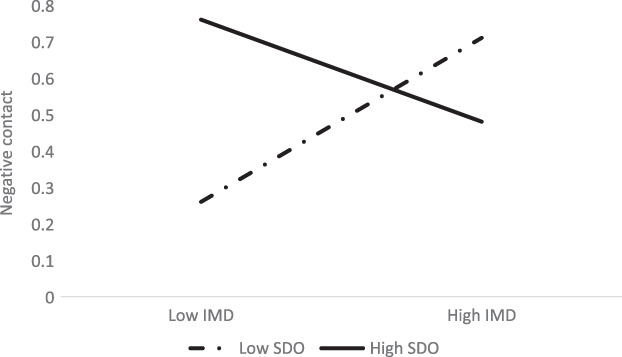
Interaction between multiple deprivation (IMD) and SDO on negative contact of White people.

The interaction between IMD and SDO was negatively associated with negative contact (Hypothesis 6; see Table 2). However, the simple slope analyses revealed no significant association between IMD and negative contact for both White people low in SDO, *β* = −0.02, 95% CI [−0.01, 0.02], and high in SDO, *β* = −0.13, 95% CI [−0.03, 0.00]. Inspection of the interaction patterns reveals a surprising result, since deprivation is associated with increased negative contact for White people low in SDO, but with decreased negative contact for White British people high in SDO (see Figure 3). The effects are, however, very small.

#### Asian British minority sample

We conducted the same set of regression analyses for the Asian British minority group sample (see Tables 4 and 5). In this sample, only one antecedent was reliably associated with contact: perceived ingroup norms were positively associated with the frequency of positive contact of Asian respondents with White respondents (Hypothesis 3). Other variables were unrelated to either positive or negative intergroup contact of Asian respondents.

**TABLE 4:** Regression analysis: Summary of factors predicting positive contact of Asian people with White people

	Step 1	Step 2a (RWA)	Step 2b (SDO)
Variable	β	β	β
Age	−0.09	−0.10	−0.08
Education	0.08	0.06	0.08
Percentage of Whites	−0.07	−0.05	−0.09
IMD	−0.01	0.01	−0.02
SDO	−0.02	−0.02	−0.03
RWA	0.10	0.09	0.09
Perceived ingroup norms	0.26***	0.26**	0.27***
Percentage of Whites × ideology		−0.11	−0.14
IMD × ideology		−0.12	−0.08
Ingroup norms × ideology		0.05	−0.07
*R* ^2^	.04		
Adjusted *R* ^2^	.12		
*F* for change in *R* ^2^	3.16*	2.73**	2.79**

*Note*: Step 1 includes all main variables; Step 2a includes RWA as ideology in the interaction terms; and Step 2b includes SDO as ideology in the interaction terms.

IMD = index of multiple deprivation; RWA = right‐wing‐authoritarianism; SDO = social domination orientation.

* *p* < .05.

** *p* < .01.

*** *p* < .001.

**TABLE 5:** Regression analysis: Summary of factors predicting negative contact of Asian people with White people

	Step 1	Step 2a (RWA)	Step 2b (SDO)
Variable	β	β	β
Age	−0.11	−0.11	−0.11
Education	0.08	0.05	0.09
Percentage of Whites	−0.01	−0.00	−0.00
IMD	−0.02	−0.01	−0.03
SDO	0.04	0.02	−0.01
RWA	0.16	0.15	0.20*
Perceived ingroup norms	0.12	0.10	0.15
Percentage of Whites × ideology		−0.09	−0.13
IMD × ideology		−0.03	0.00
Ingroup norms × ideology		0.08	−0.20*
*R* ^2^	.06		
Adjusted *R* ^2^	.02		
*F* for change in *R* ^2^	1.59	1.51	2.02*

*Note*: Step 1 includes all main variables; Step 2a includes RWA as ideology in the interaction terms; and Step 2b includes SDO as ideology in the interaction terms.

IMD = index of multiple deprivation; RWA = right‐wing‐authoritarianism; SDO = social domination orientation.

* *p* < .05.

**Figure 4:**
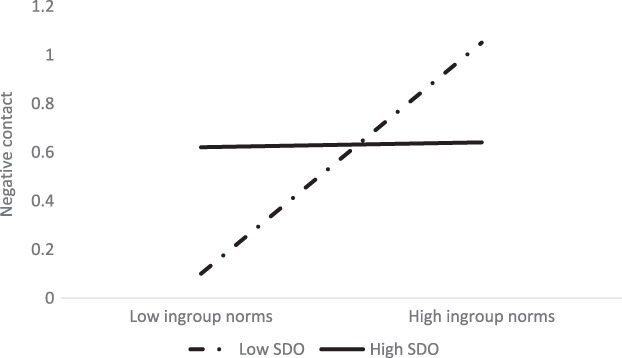
Interaction between perceived ingroup norms and SDO on negative contact of Asian people.

The product of perceived ingroup norms in favour of intergroup contact and SDO was negatively associated with negative contact (Hypothesis 7). Simple slopes (see Figure 4) showed that perceived ingroup norms were positively associated with negative contact of Asian people low in SDO, *β* = 0.25, 95% CI [0.11, 1.06], but not for those high in SDO, *β* = 0.04, 95% CI [−0.34, 0.52]. Thus, perceived ingroup norms were positively associated with negative intergroup contact for Asian people low in SDO. The other products were non‐significant.

## DISCUSSION

This study extended previous research by providing evidence concerning the joint effects of different antecedents of intergroup contact on positive and negative encounters of White British majority and Asian British minority groups with the respective ethnic outgroup. We used a daily diary method that allowed us to record the number of daily intergroup encounters to try to obtain a more accurate estimate of reported intergroup experiences compared with the most common measure of contact (Pettigrew & Tropp, 2006). We found that different factors came to the fore in the case of majority compared with minority groups.

For the majority group, results were quite complex. Neighbourhood diversity and ingroup norms were positively associated with positive daily intergroup experiences. Living in contexts characterised by multiple persistent forms of disadvantage was negatively associated with positive intergroup contact, whereas neighbourhood diversity and embracing an authoritarian ideology (i.e., high in RWA) were positively associated with negative experiences with minority group members. Moreover, living in neighbourhoods with a higher proportion of Asians was associated with higher frequencies of both positive and negative intergroup contact experiences, for respondents high in RWA. The social and economic deprivation of neighbourhoods also interacted with SDO. But simple slopes analyses revealed only small and non‐significant associations both for respondents low and high in SDO with negative contact.

For the minority group, we only found evidence of one significant antecedent of contact: perceived ingroup norms were positively associated with positive intergroup contact. These also moderated the relationship between SDO and negative intergroup contact. We discuss these findings in terms of their methodological, theoretical and practical implications, acknowledging some limitations and areas for future research as we do so.

### Methodological, theoretical and practical implications

The present research addresses urgent research questions about factors that may encourage or stifle intergroup contact (Paolini et al., 2018). In doing so, we employed the diary method, rarely used in the intergroup contact literature, allowing us to obtain a more reliable, and less subject to memory bias measure compared with the conventional measure of contact based on the recall of intergroup experiences across a longer period of time (presumably more subject to forgetting).

In regard to the drivers of intergroup contact included in our research, we supported and expanded the literature in five main ways. First, among the majority group, our findings showed that the percentage of Asian British people in UK neighbourhoods, or the psychological reality of living in a de‐segregated context that provides opportunities for ethnic majority–minority contact, was associated with increased overall actual intergroup contact. Interestingly, neighbourhood diversity was the only factor positively associated with both types of daily intergroup contact. This evidence provided a potential explanation of the mixed results on the relationship between diversity and interethnic contact (Kros & Hewstone, 2020; Laurence et al., 2018). Facilitating both positive and negative face‐to‐face intergroup contact, living close to different ethnic groups may be related to either an improvement or deterioration of intergroup relations depending on which type of contact plays a stronger role in shaping future interactions. Other intervening factors may help us to understand when neighbourhood diversity affects majority group members' tolerance and openness to positive rather than negative intergroup experiences (e.g., historical intergroup relationships, personality factors).

Second, findings related to the majority group extend the literature on the relationship between social and economic deprivation and interethnic contact (Sampson et al., 1997) by showing that respondents' level of deprivation was negatively associated with positive intergroup experiences in the case of the majority group. This evidence suggests that people who feel socially and economically threatened by ethnic minorities tend to have less positive contact with them, reducing the chances of changing their view.

Third, with regard to the literature on ideological attitudes and interethnic relationships (Cohrs & Stelzl, 2010), RWA, but not SDO, was associated with daily negative intergroup contact among members of the majority group. This finding implies that it is the authoritarianism component (tapped by RWA), more than the support for intergroup inequalities (tapped by SDO), that is linked to negative experiences with outgroup members. From the present data, we cannot be sure if this difference associated with RWA is based on the avoidance of contact by those higher in RWA or on an increased willingness to engage in contact by those lower in RWA. However, the higher frequency of both forms of valenced contact reported by those higher in RWA living in mixed areas indicates that the reduced difference of negative contact between those low and high in RWA living in the segregated areas is likely to be due to those higher in RWA avoiding contact. In this regard, although people with higher scores on right‐leaning ideologies might avoid ethnic minority outgroups, the more contact they have with these groups, the more tolerant and open‐minded their attitudes about the groups become (e.g., Hodson et al., 2013). Thus, our correlational evidence may support not just the role of ideologies in shaping intergroup experiences, but also the role of negative intergroup contact in reinforcing intolerant ideologies.

Fourth, in contrast to the potentially critical role of deprivation and ideologies, ingroup norms were associated with the likelihood of having positive, but not negative, contact with outgroup members for the majority and the minority group. Previous research consistently showed the beneficial effect of social norms in promoting positive intergroup relationships from the perspective of the majority (Christ et al., 2014). The present study included both majority and minority samples, and confirmed the importance of ingroup members as sources of information about social attitudes. Future studies should address whether specific social norms are predictive of actively seeking out negative encounters. The beneficial role of ingroup norms in encouraging only positive intergroup contact across both groups implies the relevance of this factor in implementing interventions to build an inclusive society. Even in globalised and increasingly multicultural societies that offer growing opportunities for intergroup contact, people may still avoid engaging in meaningful experiences with outgroup members (Dixon & Durrheim, 2003). Thus, favouring ingroup norms in support of intergroup contact may be a strategic way to implement social integration.

Fifth, we examined the interplay between the various factors considered. We extended previous research on the role of diversity on positive intergroup contact (Brune et al., 2016) by showing that White British people who endorse authoritarian ideologies tend to avoid intergroup encounters in general and thereby not only report less positive but also less negative intergroup contact than White British people who agree less with authoritarian ideologies. However, when intergroup contact becomes unavoidable, high compared with low RWA people increased both their positive and negative experiences with outgroup members. Therefore, our findings are encouraging in supporting the role of neighbourhood diversity in improving intergroup encounters even for more authoritarian people, who tend to be more prejudiced. We thus extended previous research (Brune et al., 2016) by showing that White British people who endorsed authoritarian ideologies tended to avoid not just positive but also negative intergroup contact when they had the chance to do so (e.g., when the percentage of Asian British people living in their neighbourhood was low). Social diversity, rather than ingroup norms, plays a crucial role in increasing intergroup contact for high authoritarians, people who tend to be more prejudiced. This can be particularly relevant for interventions with prejudiced people when seeking to promote the inclusion of immigrants as well as their adaptation into host societies.

Our exploratory analyses on the interplay between different predictors of intergroup contact showed that among minority group members those low in SDO who perceived higher ingroup norms in favour of intergroup contact reported more frequent negative experiences with the majority group. This surprising finding supports the link between ingroup norms and intergroup experiences. It implies that especially minority group members who both embrace ingroup norms concerning intergroup contact and are less authoritarian are those who tend to have more intergroup contact, including a higher frequency of negative experiences, with the majority group.

Overall, the factors we investigated were differentially related to daily positive and negative intergroup experiences, highlighting the importance of considering both forms of valenced contact in future research to further understand when and how contact can improve intergroup relationships (Schäfer et al., 2021). Going beyond the optimal conditions proposed by Allport (1954), we found that a mix of key factors, including perceived ingroup norms and social diversity, especially for authoritarians, can be the catalysts for virtuous cycles of contact and further contact‐seeking for majority and, in part also, for minority groups members.

Notwithstanding the novel findings of this research, and consistent results across measures, we acknowledge some limitations. First, the data are correlational, so we cannot draw any conclusions about the direction of causation. Second, there was a high drop‐out rate, preventing us from using all 13 days of diary data. Given the challenge of collecting diary data from a large community sample, including minority members, the agency we contracted was committed to reach at least six completed diary entries for at least 150 respondents of each sample, and this might have been linked to the significant reduction of responses after day five of the diary. Third, we acknowledge the low internal consistency for the RWA short scale. However, this low reliability is to be expected since the short scale captures all three sub‐dimensions of RWA, and measures of internal consistency are inapplicable in the case of heterogeneous constructs (e.g., McNeish, 2018). We were not able to provide a proper reliability estimate like omega for heterogeneous measures, since the short scale contains only one item for each sub‐dimension of RWA.

## CONCLUSION

This novel, diary‐based study investigated antecedents of both majority White British and minority Asian British respondents' valenced intergroup contact. We found that different factors—at different levels—were related to positive and negative intergroup contact for members of the two groups. For the majority group, neighbourhood diversity (macrolevel) was positively associated with both positive and negative forms of contact, whereas social and economic deprivation of neighbourhoods was negatively associated with positive contact; ingroup norms in favour of contact with the outgroup (mesolevel) were positively associated with positive intergroup encounters; and Right Wing Authoritarianism (microlevel) was positively associated with negative intergroup contact. For the minority group, however, only ingroup norms (mesolevel) were positively associated with positive intergroup contact. We also found evidence from exploratory analyses for some more complex interaction effects between the antecedents, with micro‐level factors (SDO and RWA) as moderators. Interestingly, norms were the only antecedent to emerge for both groups—ingroup norms were associated with the likelihood of having positive, but not negative, contact with outgroup members –and we highlighted its potential for future interventions. Overall, these findings highlight the value of studying antecedents of contact separately for majority and minority groups, for both positive and negative forms of contact, and of taking a broad approach, encompassing macro‐, meso‐ and microlevel factors.

## Supporting information


**Appendix**.

## Endnote

^1^
Cronbach's alphas are: Social Dominance Orientation (White *α* = .75; Asian *α* = .63), Right‐Wing‐Authoritarianism (White *α* = .58; Asian *α* = .55), perceived ingroup norms (White *α* = .87; Asian *α* = .86). However, Cronbach's alpha is rarely an appropriate measure of reliability and omega is an alternative valid measure (McNeish, 2018).

## References

[ijop12841-bib-0001] Al Ramiah, A. A. , Schmid, K. , Hewstone, M. , & Floe, C. (2015). Why are all the white (Asian) kids sitting together in the cafeteria? Resegregation and the role of intergroup attributions and norms. British Journal of Social Psychology, 54(1), 100–124. 10.1111/bjso.12064 24597949

[ijop12841-bib-0002] Allport, G. W. (1954). The nature of prejudice. Addison‐Wesley.

[ijop12841-bib-0003] Altemeyer, B. (1991). Right wing authoritarian scale. In J. P. Robinson , P. R. Shaver , & L. S. Wrightsman (Eds.), Measures of personality and social psychological attitudes (Vol. 1, pp. 550–555). Academic Press.

[ijop12841-bib-0004] Barlow, F. K. , Paolini, S. , Pedersen, A. , Hornsey, M. J. , Radke, H. R. , Harwood, J. , Rubin, M. , & Sibley, C. G. (2012). The contact caveat: Negative contact predicts increased prejudice more than positive contact predicts reduced prejudice. Personality and Social Psychology Bulletin, 38(12), 1629–1643. 10.1177/0146167212457953 22941796

[ijop12841-bib-0005] Beierlein, C. , Asbrock, F. , Kauff, M. , & Schmidt, P. (2014). Die Kurzskala Autoritarismus (KSA‐3): Ein ökonomisches Messinstrument zur Erfassung dreier Subdimensionen autoritärer Einstellungen [A short scale assessing authoritarianism: A parsimonious scale to assess three subdimensions of authoritarian attitudes] (GESIS Working Papers 2014/35). http://nbn‐resolving.de/urn:nbn:de:0168ssoar‐426711

[ijop12841-bib-0006] Brune, A. , Asbrock, F. , & Sibley, C. G. (2016). Meet your neighbours. Authoritarians engage in intergroup contact when they have the opportunity. Journal of Community and Applied Social Psychology, 26(6), 567–580. 10.1002/casp.2289

[ijop12841-bib-0007] Christ, O. , Schmid, K. , Lolliot, S. , Swart, H. , Stolle, D. , Tausch, N. , Al Ramiah, A. , Wagner, U. , Vertovec, S. , & Hewstone, M. (2014). Contextual effect of positive intergroup contact on outgroup prejudice. PNAS, 111(11), 3996–4000. 10.1073/pnas.1320901111 24591627 PMC3964129

[ijop12841-bib-0008] Cohrs, J. C. , & Stelzl, M. (2010). How ideological attitudes predict host society members' attitudes toward immigrants: Exploring cross‐national differences. Journal of Social Issues, 66(4), 673–694. 10.1111/j.1540-4560.2010.01670.x

[ijop12841-bib-0009] Dhont, K. , & van Hiel, A. (2009). We must not be enemies: Interracial contact and the reduction of prejudice among authoritarians. Personality and Individual Differences, 46(2), 172–177. 10.1016/j.paid.2008.09.022

[ijop12841-bib-0010] Dixon, J. , & Durrheim, K. (2003). Contact and the ecology of racial division: Some varieties of informal segregation. British Journal of Social Psychology, 42(1), 1–23. 12713753 10.1348/014466603763276090

[ijop12841-bib-0011] Gómez, A. , Tropp, L. R. , & Fernández, S. (2011). When extended contact opens the door to future contact: Testing the effects of extended contact on attitudes and intergroup expectancies in majority and minority groups. Group Processes & Intergroup Relations, 14(2), 161–173. 10.1177/1368430210391119

[ijop12841-bib-0012] Graf, S. , Paolini, S. , & Rubin, M. (2014). Negative intergroup contact is more influential, but positive intergroup contact is more common: Assessing contact prominence and contact prevalence in five central European countries. European Journal of Social Psychology, 44(6), 536–447. 10.1002/ejsp.2052

[ijop12841-bib-0013] Heller, A. , Decker, O. , Schmalbach, B. , Beutel, M. , Fegert, J. M. , Brähler, E. , & Zenger, M. (2020). Detecting authoritarianism efficiently: Psychometric properties of the screening instrument authoritarianism–ultra short (A‐US) in a German representative sample. Frontiers in Psychology, 11, 533863. 10.3389/fpsyg.2020.533863 33329171 PMC7732445

[ijop12841-bib-0014] Hodson, G. , Costello, K. , & MacInnis, C. C. (2013). Is intergroup contact beneficial among intolerant people? Exploring individual differences in the benefits of contact on attitudes. In G. Hodson & M. Hewstone (Eds.), Advances in intergroup contact (pp. 49–80). Psychology Press.

[ijop12841-bib-0015] Jetten, J. , Spears, R. , & Manstead, A. S. (1996). Intergroup norms and intergroup discrimination: Distinctive self‐categorization and social identity effects. Journal of Personality and Social Psychology, 71(6), 1222–1233. 8979388 10.1037//0022-3514.71.6.1222

[ijop12841-bib-0016] Kauff, M. , Beneda, M. , Paolini, S. , Bilewicz, M. , Kotzur, P. , O'Donnell, A. W. , Stevenson, C. , Wagner, U. , & Christ, O. (2021). How do we get people into contact? Predictors of intergroup contact and drivers of contact seeking. Journal of Social Issues, 77(1), 38–63. 10.1111/josi.12398

[ijop12841-bib-0017] Kotzur, P. F. , Tropp, L. R. , & Wagner, U. (2018). Welcoming the unwelcome: How contact shapes contexts of reception for new immigrants in Germany and the United States. Journal of Social Issues, 74(4), 812–832. 10.1111/josi.12300

[ijop12841-bib-0018] Kros, M. , & Hewstone, M. (2020). Negative and positive interethnic contact and the consequences of ethnic neighborhood composition for trust, cohesion, and prejudice. European Sociological Review, 36(6), 937–956. 10.1093/esr/jcaa032

[ijop12841-bib-0019] Laurence, J. (2014). Reconciling the contact and threat hypotheses: Does ethnic diversity strengthen or weaken community inter‐ethnic relations? Ethnic and Racial Studies, 37(8), 1328–1349.

[ijop12841-bib-0020] Laurence, J. , Schmid, K. , & Hewstone, M. (2018). Ethnic diversity, inter‐group attitudes and countervailing pathways of positive and negative inter‐group contact: An analysis across workplaces and neighborhoods. Social Indicators Research, 136(2), 719–749. 10.1007/s11205-017-1570-z 29563660 PMC5842268

[ijop12841-bib-0021] McNeish, D. (2018). Thanks coefficient alpha, we'll take it from here. Psychological Methods, 23(3), 412–433. 10.1037/met0000144 28557467

[ijop12841-bib-0022] Office for National Statistics . (2013). 2011 census: Ethnic group, local authorities in the United Kingdom. http://www.ons.gov.uk/ons/rel/census/2011‐census/key‐statistics‐and‐quick‐statistics‐for‐local‐authorities‐in‐the‐united‐kingdom‐‐‐part‐1/rft‐ks201uk.xls

[ijop12841-bib-0023] Paolini, S. , Harwood, J. , Hewstone, M. , & Neumann, D. L. (2018). Seeking and avoiding intergroup contact: Future frontiers of research on building social integration. Social and Personality Psychology Compass, 12(12), e12422. 10.1111/spc3.12422

[ijop12841-bib-0024] Pettigrew, T. , & Tropp, L. (2006). A meta‐analytic test of intergroup contact theory. Journal of Personality and Social Psychology, 90(5), 751–783. 10.1037/0022-3514.90.5.751 16737372

[ijop12841-bib-0025] Pratto, F. , Cidam, A. , Stewart, A. L. , Zeineddine, F. B. , Aranda, M. , Aiello, A. , Chryssochoou, X. , Cichocka, A. , Cohrs, J. C. , Durrheim, K. , Eicher, V. , Foels, R. , Górska, P. , Lee, I.‐C. , Licata, L. , Liu, J. , Liu, L. , Meyer, I. , Davide, M. , & Eicher, V. (2013). Social dominance in context and in individuals: Contextual moderation of robust effects of social dominance orientation in 15 languages and 20 countries. Social Psychological and Personality Science, 4(5), 587–599. 10.1177/1948550612473663

[ijop12841-bib-0026] Sampson, R. J. , Raudenbush, S. W. , & Earls, F. (1997). Neighborhoods and violent crime: A multilevel study of collective efficacy. Science, 277(5328), 918–924. 10.1126/science.277.5328.918 9252316

[ijop12841-bib-0027] Schäfer, S. J. , Kauff, M. , Prati, F. , Kros, M. , Lang, T. , & Christ, O. (2021). Does negative contact undermine attempts to improve intergroup relations? Deepening the understanding of negative contact and its consequences for intergroup contact research and interventions. Journal of Social Issues, 77(1), 197–216. 10.1111/josi.12422

[ijop12841-bib-0028] Sidanius, J. , & Pratto, F. (1999). Social dominance: An intergroup theory of social hierarchy and oppression. Cambridge University Press.

[ijop12841-bib-0029] Social Mobility Commission . (2016). Ethnicity, gender and social mobility. https://www.gov.uk/government/uploads/system/uploads/attachment_data/file/579988/Ethnicity_gender_and_social_mobility.pdf.

[ijop12841-bib-0030] van Assche, J. , Roets, A. , Dhont, K. , & van Hiel, A. (2016). The association between actual and perceived ethnic diversity: The moderating role of authoritarianism and implications for outgroup threat, anxiety, and mistrust. European Journal of Social Psychology, 46(7), 807–817. 10.1002/ejsp.2211

