# Supplementary material for: Antecedents of positive and negative intergroup contact: Evidence from a diary study
Source: Int J Psychol. 2022 Mar 8;57(4):524–34. doi: 10.1002/ijop.12841 (PMC10286651; doi:10.1002/ijop.12841)
Supplement: Supplementary file 1 — Appendix. [file IJOP-57-524-s001.docx]

**Appendix**

**Data Availability:** Bracegirdle, C., Hewstone, M., Schmid, K., Schäfer, S. J., Christ, O., Jaspers, E., … Prati, F. (2020, March 9). UK Diary Study. Retrieved from osf.io/fj2m7

**Figure A.1.**

Frequencies of White British people’s positive daily contact.

**Figure A.2.**

Frequencies of White British people’s negative daily contact.

**Figure A.3.**

Frequencies of Asian British people’s positive daily contact.

**Figure A.4.**

Frequencies of Asian British people’s negative daily contact.

**Table A.1.**

Attrition for White British and Asian British respondents (those who completed both positive and negative diary measures from Day 1 to Day 5 vs. missing)

|  | White British sample | | | Asian British sample | | |
| --- | --- | --- | --- | --- | --- | --- |
|  | Respondents  (*n* =499) | Missing  (*n* =245) | Significant difference and  Cohen’s *d* | Respondents  (*n* =199) | Missing  (*n* =383) | Significant difference and Cohen’s *d* |
| Age | 49.29 | 43.31 | * (-0.40) | 46.12 | 39.49 | * (-0.48) |
| Income | 5.96 | 5.70 | - | 6.18 | 5.95 | - |
| Education | 3.72 | 4.06 | * (0.23) | 3.92 | 4.28 | * (0.24) |
| Percentage of Asians | 9.84 | 14.54 | * (0.25) |  |  |  |
| Percentage of White |  |  |  | 83.11 | 64.99 | * (-0.70) |
| IMD | 20.31 | 21.74 | - | 22.27 | 25.24 | - |
| SDO | 2.05 | 2.02 | - | 2.03 | 2.09 | - |
| RWA | 3.05 | 2.94 | - | 3.01 | 3.16 | * (0.18) |
| Perceived ingroup norms | 3.61 | 3.66 | - | 3.72 | 3.84 | - |

*Note*. For White British sample: Little’s MCAR test: Chi-Square = 860.230, DF = 817, *p* = .143; for Asian British sample: Little’s MCAR test: Chi-Square = 220.874, DF = 113, *p* < .001 (considering: gender, age, income, education, percentage of Asians, IMD, SDO, RWA, perceived ingroup norms). IMD = Index of Multiple Deprivation; SDO = Social Domination Orientation; RWA = Right-Wing-Authoritarianism.
